# Demand of elderly people for residential care: an exploratory study

**DOI:** 10.1186/1472-6963-6-39

**Published:** 2006-03-27

**Authors:** PMA van Bilsen, JPH Hamers, W Groot, C Spreeuwenberg

**Affiliations:** 1 Department of Health Care Studies, Section Nursing Science, Universiteit Maastricht, P.O. Box 616, 6200 MD Maastricht, The Netherlands; 2 Department of Health Organization, Policy and Economics, Universiteit Maastricht, Maastricht, The Netherlands; 3 Department of Health Care Studies, Universiteit Maastricht, Maastricht, The Netherlands

## Abstract

**Background:**

Because of the rapid aging population, the demand for residential care exceeds availability. This paper presents the results of a study that focuses on the demand of elderly people for residential care and determinants (elderly people's personal characteristics, needs and resources) that are associated with this demand. Furthermore, the accuracy of the waiting list as a reflection of this demand has been examined.

**Methods:**

67 elderly people waiting for admission into a home for the elderly, are subjected to semi-structured interviews. The data are analyzed by using multivariate statistics.

**Results:**

Elderly people who indicate that they would refuse an offer of admission into a home for the elderly feel healthier (p = 0.02), have greater self-care agency (p = 0.02) and perceive less necessity of admission (p < 0.01), compared to those who would accept such an offer. Especially the inability to manage everyday activities and the lack of a social network are highly associated with the elderly people's demand for residential care. Furthermore, it is evident that waiting lists for homes for the elderly do not accurately reflect the demand for residential care, since 35% of the elderly people on a waiting list did not actually experience an immediate demand for residential care and stated that they would not accept an offer of admission. Quite a lot of respondents just registered out of a sense of precaution; a strategic decision dictated by current shortages in care provision and a vulnerable health status.

**Conclusion:**

The results contribute to the understanding of waiting lists and the demand for residential care. It became apparent that not everybody who asks for admission into a home for the elderly, really needed it. The importance of elderly people's resources like social networks and the ability to manage everyday activities in relation to the demand for care became clear. These findings are important because they indicate that resources also play a role in predicting elderly people's demand and as a result can guide the development and the (re)design of adequate health care services.

## Background

In most Western countries, the health care system faces serious problems as a result of social trends and advances in medicine such as the ageing of the population, changes in the provision of care, increase in well-informed patients and predictive medical information. These changes have profound impact on the nature and the extent of health care services for elderly people. On the one hand, the ageing of the population already has led to staffing shortages in care and delay in capacity expansion [1-4]. For example, in the Netherlands 33.029 elderly persons were waiting for admission into a home for the elderly in November 2002 [5] showing that demand exceeds availability resulting in waiting-lists. On the other hand, elderly people increasingly want and demand a more varied provision of health care services at home so they can age in their familiar home environment as long as possible [4,6-12]. Therefore, policy-makers and researchers argue that waiting-lists possibly do not adequately reflect elderly people's demands an as a result do not adequately reflect the services elderly prefer. Consequently, this raises the question what services should be offered in order to meet the demand for health care of elderly people?

**Figure 1:**
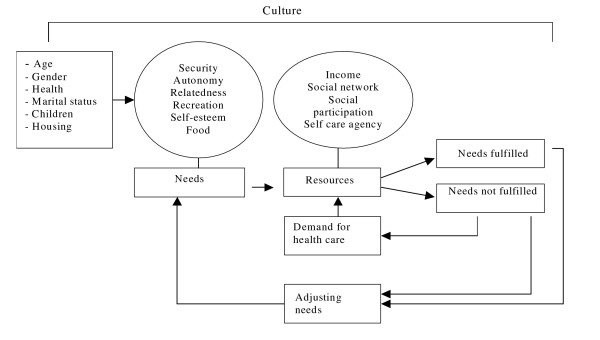
Relation between health care needs, resources and demands.

To answer this question, several studies [e.g [1,9-11,13,14]] have been reviewed which resulted in the development of a theoretical model on the relationship between elderly people's needs, resources and their demand for care (see Figure 1). A major assumption in this model is that people address universal needs (e.g. autonomy, self-esteem) and that these needs can be influenced by a range of personal characteristics (e.g. age, health status, cognitive status). Besides, people attribute a prioritisation of relative importance to these universal needs dependent on culture, person-bound characteristics of people (e.g. age, gender, social class) and changing circumstances (e.g. household composition) [15-18]. The second major assumption is that people use resources like self-care agency, income, informal care and a social network to fulfil their needs. A demand for care is a result of one or more unmet needs, mainly due to insufficiencies in available resources. This is the third major assumption in the model.

The aim of this study is get insight into the determinants of the demand for residential care. The model serves as a framework to study these determinants and related factors. In addition, the question whether waiting lists do accurate reflect the elderly people's demands, is evaluated. This study focuses on elderly people on a waiting list for a home for the elderly in the Netherlands. Homes for the elderly are institutions providing living conditions for old people who are unable to live independent alone, but usually require no more nursing care than can be given by a visiting nurse [19]. Elderly people in the Netherlands on a waiting list, previously made an official request for residential care at an assessment agency's office. These agencies are established in order to control the growing demand by formalizing and standardizing the process of determining the patient's needs for the health services to which they are entitled [20,21]. Without approval of these assessment offices, the caregiver is not allowed to provide care. The central questions in this study are:

- What determinants are associated with the demand for admission into a home for the elderly?

- Are waiting lists for admission into a home for the elderly an accurate reflection of the demands of elderly people for services in later life?

## Methods

A cross-sectional design was used. Over a period of two months elderly persons who were registered for admission into a home for the elderly were interviewed.

Health insurance agencies in Limburg (a province in the south of the Netherlands) were asked to randomly select 200 elderly people registered on a waiting list for a home for the elderly. At that moment, there were 1693 elderly people waiting for admission into a home for the elderly in Limburg [5]. The mailing list consist of 197 addresses of elderly people (3 addresses were removed because of incompleteness). Subsequently, a letter was sent informing the recipients about this study. A few days after this letter had been sent, the subjects were contacted by telephone to ask whether they would like to participate. If they agreed, a date was set for an interview. Reasons for refusing were systematically recorded. Eleven interviewers, who had been carefully instructed, interviewed the subjects at home. All interviews were taped and fully transcribed.

A considerable part of the 197 selected elderly persons could not be reached by phone or were no longer on the waiting list (see Table 1). Of the remaining prospective participants (n = 163), 22 were never reached because there was no answer even though we kept trying for a lengthy period of time, and 50 elderly people showed no interest. These 50 people were not convinced of the usefulness of the research, were too busy with other things (e.g. their social life, home improvement), were afraid of the consequences of cooperation (being removed from a waiting list or the opposite being offered a place in a home) or were tired of all the interviews and consultations they had already had (e.g. with home care organisations, regional assessment agencies, insurance companies). Another 26 elderly people claimed to be too sick, confused or disabled (e.g. because of hearing problems). Eventually, 65 interviews were conducted. Based on the 163 respondents that were reached by phone, the response was thus 40%. It subsequently appeared that 3 interviews could not be used because they had too many missing values. So 62 interviews were analysed, involving 57 singles and 5 couples. The answers of the couples were analyzed separately, so the results relate to the answers of 67 respondents. For an overview of the response and non-response see Table 1.

Complementary quantitative and qualitative data were collected by means of interviews. A semi-structured questionnaire served as a guideline. This questionnaire was developed based on the theoretical model on the relationship between needs, resources and demands (See Figure 1) [11]. Questions were asked about the following subjects:

- Personal characteristics (e.g. age, gender, marital status, children, housing)

- Health status (functional status, subjective perception of health, loneliness, memory loss). Functional status was measured by the Groningen Activity Restriction Scale [22]. Respondents were asked to indicate to what extent they were still able to engage in activities of daily living related to personal care (11 activities) and activities related to independent living (7 activities). The respondent had to answer on a 3-point ordinal scale to what extent they can carry out the activities of daily living either independently or (partly) with help of others (1 = completely independent, 2 = independent with great effort, 3 = only with help of others).

The Scale of Subjective Welfare was used to measure the subjective perception of health status (5 items), the existence of negative feelings (5 items) and feelings of loneliness (7 items) [23]. The respondents had to answer on a 3-point ordinal scale to what extent they agreed (0 = disagree, 1 = do not know, 2 = agree) with statements, such as 'I feel fine', 'I feel quite healthy', 'I worry a lot', 'I often feel sad', 'I miss a really good friend', 'I experience an inner void'. Analogous to these items scored on a 3-point ordinal scale to what extent they agreed, three self developed questions were added concerning loss of memory, like 'I have to write down everything I need to remember', 'I am very forgetful' and 'I do not suffer from memory loss'. Higher scores indicated greater perception of being in good health, having more memory problems, having more negative feelings (sadness, anxiety, worries) or greater loneliness.

- Presence of resources like social network, social participation, income and self-care agency. Social network was measured by asking whether the respondents were satisfied with regard to the frequency in which they see their children (1 item). Next, 4 items were formulated about the existence of satisfactory contacts with friends, neighbours, family. The respondents had to answer on a 5-point ordinal scale to what extent they agreed with the statements, such as 'I have a lot of close friends' and 'I have a circle of friends on which I can rely when sudden problems arise' (1 = I strongly disagree, 2 = I disagree, 3 = neither agree or disagree, 4 = I agree, 5 = I strongly agree). A high score on the self-developed 'social network' items meant that respondents had satisfactory contacts with their children and friends and felt they could rely on them. Another 4 questions measured the extent to which the elderly persons still participated in social life. These self-developed items also had to be answered on a 5-point ordinal scale to what extent they agreed. For example, 'I still do voluntary work', 'I'm still a member of more then one social club'. A higher score indicated that the respondent had a satisfactory social life and was still involved in social activities.

Two self-developed items were added about the sufficiency of their income. The respondents had to answer these items yet again on a 5-point ordinal scale to what extent they agreed with the statements posed. For example, 'I have enough income to arrange my life the way I wanted to be', 'I cannot buy everything that I need'. A higher score indicated that the respondent experienced their income as sufficient.

Self-care agency was measured by 9 items of the ASA-A [24]. Self-care agency is defined as the respondents' capability to search for and obtain continuous care for themselves. Respondents were also asked about their ability to live and eat in a healthy manner. The respondents had to respond to these statements on a 5-point ordinal scale to indicate the extent to which they agreed or disagreed. Higher scores meant greater self-care agency (1 = I strongly disagree, 2 = I disagree, 3 = neither agree or disagree, 4 = I agree, 5 = I strongly agree).

- Demand for residential care. The demand for admission into a home for the elderly was assessed by asking three questions. The first question inquired if they would today accept or refuse an offer to move into a home for the elderly not of their first choice. The second, asked if they would today accept or refuse an offer to move into the home of their first choice. The third question asked the respondents to rate the necessity of admission into a home for the elderly on a 5-point ordinal scale (1 = not at all necessary, 2 = not necessary, 3 = neither necessary nor unnecessary, 4 = necessary, 5 = very necessary).

Besides, four questions were asked about the registration on a waiting list and motives underlying this registration. The respondents were asked if they were on a waiting list for residential care and how long they were registered. The duration of registration was also checked with the formal registration of the administrators. Then the respondents were questioned about their motives underlying their choice to register for residential care by means of an open question. Subsequently, they had to select the motive of most importance.

Table 2 summarises the characteristics of some of the above mentioned measures (Cronbach's α, range, sum scores, deviation). In general, the measurement instruments proved to be internally consistent (Cronbach's α range based on the data is between 0.66 and 0.89) [25].

Besides descriptive statistics (frequencies, mean, deviation, sum scores, Cronbach's α) the data were analysed by comparing groups (One-way ANOVA and chi square); respondents who indicated they would accept an offer of admission were compared with those who said they would refuse such an offer, using multivariate statistics (regression analysis). The interviews with the elderly were fully transcribed, allowing the answers ticked by the interviewers to be verified. The interview transcripts also allowed us to gather more detailed information about aspects like the underlying motives to move into a home for the elderly.

## Results

### Sample

The sample included 50 female and 17 male respondents (57 singles and 5 couples). The average age was 81 years (SD = 6, range 55–94). Fifty-three respondents lived on their own while 14 respondents lived together with someone else: partner (n = 12), children (n = 1), others (n = 1). Forty-eight respondents were widowed, 12 married, 2 divorced and 5 respondents were never married. Nearly all of them had children (n = 60). Twenty-seven respondents were living in a non-ground floor apartment or regular house with a first and second floor (without a lift to get to the second floor). Twenty-one respondents were living in single-store houses with only ground-floor rooms but no other adaptations. Thirteen respondents already moved to a ground-floor apartment fully adapted for elderly people. Finally, one respondent temporarily resided in a home for the elderly.

### Health

Seven respondents did not answer the questions on functional status because they wished to stop the interview at this point. The results show that the other 60 respondents needed assistance from others particularly for activities requiring a great deal of physical effort, like certain household activities, going up and down the stairs, moving outside the house, grocery shopping, preparing food and doing and ironing the laundry. Except for 'washing and drying your entire body' and 'looking after your feet', the respondents were still able to manage their personal care themselves. Those who did need help with personal care predominantly received aid from formal caregivers. In addition to formal caregivers, relatives usually assisted in house activities like grocery shopping and doing and ironing the laundry.

Thirty-three respondents perceived their health as mainly negative (score ≤ 1) (subjective perception of health status). The other responding elderly (n = 26) were more positive about their health and more likely to confirm that they were feeling fine (score>1). The results for loss of memory show that there were two almost equally sized groups, one comprising respondents (n = 31) who were annoyed by their (nascent) loss of memory (score>1) and one comprising respondents (n = 27) who reported that they did not experience any memory problems (score ≤ 1). The first group indicated that they were very forgetful and needed to write everything down to remember. Forty-three respondents reported that they had a generally positive attitude to life, meaning that they were usually not sad, worried or anxious (score ≤ 1) (negative feelings). Twenty-six respondents did experience feelings of loneliness (score>1), meaning that they felt left alone and really missed a good friend or the company of others.

### Presence of resources

Thirty-nine respondents stated that they saw their children frequently enough. Thirty-four elderly saw their children at least a few times a week. The analyses showed that 20 respondents did not have a social network or only a very small one (score ≤ 3). Nineteen elderly had a neutral score in this respect meaning that they did have some friends and children living nearby, but did not feel it was possible to fall back on them when sudden problems arose. Only 5 respondents still participated in social life (score ≥ 4).

Forty-seven respondents had a moderate to satisfactory capability to search and obtain continuous care for themselves (self-care agency). This also included their ability to live and eat in a healthy manner. In terms of the individual items, most respondents seemed to know where to turn to for information about their health and to obtain care.

### The demand for residential care

All respondents were officially on a waiting list for residential care. Three respondents, however, were not aware of this registration with the result that two respondents refused to answer the questions concerning this subject. The other respondents (n = 65) of which 5 couples, confirmed being registered for admission into a home for the elderly. The average time on the waiting list was 20 months (SD = 15).

The respondents generally mentioned more than one motive which played a substantial part in their decision to register for admission into a home for the elderly. The average number of motives mentioned was 3 (SD = 1.8). According to twenty-two respondents (34%), the most important reason was a sense of precaution. Because of their vulnerable health and the current shortages in care provision, they wanted to ensure that care would be available by the time they needed it. Fourteen respondents (22%) stated that their physical or mental disability was of overriding importance. These respondents pointed out that they felt increasingly vulnerable living on their own. The most important motive for another 9 respondents (14%) was feelings of loneliness.

A large majority of the respondents (59 out of 65 respondents) stated that they would at present refuse an offer of admission to a home that was not their first choice. Besides, 23 respondents (out of 65) would not even accept an offer of admission into the home of their first choice, although 42 respondents would. Further, 27 respondents did not consider admission to be necessary, while 31 respondents did (7 respondents judged admission neither necessary or non-necessary). Table 3 presents the results of a comparison between the group of respondents who indicated that they would accept an offer of admission and the group who said that they would refuse such an offer. Respondents who indicated that they would refuse an offer of admission into the home of their first choice felt healthier (p = 0.02), had greater self-care agency (p = 0.02) and perceived less necessity of admission (p < 0.01), compared to those who would accept such an offer. The two groups also differed with regard to their motives underlying their choice to register for residential care. Respondents who would refuse an offer were more likely to be on the waiting list because of a sense of precaution than those who would not refuse such an offer (p < 0.01). Respondents who mentioned 'feelings of loneliness' or 'health problems' as the most important motive for wanting to move into a home for the elderly were more likely to accept an offer than those who did not mention one of these reasons as the most important one.

### Factors associated with the demand for admission into a home for the elderly

Because of the limited number of respondents, it was not possible to include all variables in the regression model in order to predict the demand for admission into a home for the elderly. Therefore, the variables were first explored univariately to ascertain whether a linear model was appropriate for describing the relationship and to identify possible outliers that might distort results. Then, five independent variables – age, social network, personal perception of health status, level of physical limitation, self-care agency – were included in the regression model. The dependent variable was the acceptance or refusal of an offer to be admitted into a home for the elderly ('yes, I would accept such an offer', 'no, I would refuse such an offer').

The regression analysis showed that respondents who would immediately accept an offer of admission had a limited social network and experience more physical limitations in everyday activities (see Table 4). The more satisfactory the social network (defined as having good relationships with children, neighbours and relatives, and feeling provided for if sudden problems occur), the smaller the chance (OR 0.80; 95% CI 0.65–0.98) that they would accept such an offer. In terms of the extent of physical limitations, it was found that the chance of acceptance becomes higher as physical limitations increase (OR 1.13; 95% CI 1.00–1.27).

## Discussion and conclusion

The results of this study show that the inability to manage everyday activities and the lack of a social network are strongly associated with elderly people's demand for residential care. Furthermore, this study clearly showed that waiting lists for homes for the elderly do not accurately reflect the demand for residential care, since 35% of the elderly people on a waiting list for residential homes did not actually experience an immediate demand for this type of care and stated that they would not accept an offer of admission into the home of their first choice.

Before we discuss the findings of this study in more detail, some limitations of this study should be addressed. First, this study is explorative and employed a cross-sectional design. As a result, no definitive conclusions regarding causal relationships (predictors) can be made. Furthermore, the response rate was moderate (40%). Although this is not unusual for this specific population [26], it undoubtedly has consequences for the generalisability of the findings. The demographic characteristics of the sample in this study are comparable with those of similar populations [27] and nation wide demographics [3]. However, the possibility of selection bias cannot be ignored. It seems possible that the study covered those elderly in best health, because 26 elderly people of the 163 prospective participants refused to participate due to poor health. This also might overestimate the share of those who were on the waiting list due to a sense of precaution. Future research should pay more attention to this problem of non-response.

The results contribute to the understanding of waiting lists and the demand for residential care. It became clear that not everybody who asks for admission into a home for the elderly, really needed it. This discrepancy puts a great burden on the health care system because elderly people who really need residential care are not receiving the care they want and are entitled to. Firstly, it should be prevented that a considerable proportion elderly Dutch citizens succeed in obtaining a place on a waiting list out of a sense of precaution in spite of a thorough and bureaucratic needs assessment procedure. The assessment agencies should pay more attention to incorporating the views of the elderly people into the assessment procedure and pay explicit attention to underlying motives for registering for this type of care and available resources. Secondly, elderly people should be well informed about what to do when problems become aggravated, especially because the waiting period for admission can be quite long [26]. Thirdly, it seems worthwhile to assess the needs, availability of resources and the demands of elderly persons repeatedly even when they are still on a waiting list because these factors often fluctuate over time, due to illness, recovery or other circumstances. For instance, elderly people often opt to register for residential care after grave health problems, for example CVA or fractures due to falls. Some of them recover and then no longer perceive the need to move to a home for the elderly. Nevertheless, most of them never take action to have their names removed from the waiting list. Finally, the contamination of waiting lists should be tackled in the near future to get a more realistic idea of the numbers of people waiting for residential care. It became clear from contacting the elderly that a considerable number of elderly people registered on a waiting list were already admitted into a home for the elderly or deceased (n = 22). Another 15 could not reached due to administrative problems, very likely caused by death or moving house. This means that 19% of the elderly people we approached was in fact no longer waiting.

The results of this study also indicates that the availability of resources like social network and ability to manage daily activities play a significant role in predicting elderly people's demand for residential care. According to the model presented earlier (Figure 1) these factors could be predictors for the demand for residential care, and as a result are of importance for the development of adequate health care services. However, it should be stressed that the present study was cross-sectional. Furthermore, the study only examined a few resources in relation to the need for residential care due to a low number of cases. Therefore, future longitudinal research is needed and should focus on more cases and other possible resources in relation to the elderly people's demand for residential and other types of care, like the amount of social participation, living arrangement/housing, existence of informal care, material resources, transportation [14,28-37]. This also holds true for the operationalization of other components of the model, and relation between concepts.

## Competing interests

The author(s) declare that they have no competing interests.

## Authors' contributions

All authors critically reviewed the manuscript, read and approved the final manuscript.

## Pre-publication history

The pre-publication history for this paper can be accessed here:



## Figures and Tables

**Table 1:** Total response and overview of reasons for non-response (n = 200)

Number of subjects selected	**200**
Number of subjects approached by letter	
- 3 addresses were removed because they were incomplete	-3 197

Number of subjects not available for participation	
- 2 deceased	
- 19 admitted into homes for the elderly	
- 1 person claimed not to be on a waiting list	-22 175

Number of subjects who could not been reached by phone due to administrative problems	
- 6 phone numbers were out of order	
- 5 addresses seemed to be wrong (selected person unknown)	
- 1 person had moved out and left no forwarding address	-12 **163**

Number of subjects who could not been reached over a lengthy period of time (n = 22)	-22 141

Number of subjects who did not want to participate	
- lack of interest (n = 50)	
- being too sick, confused or disabled (n = 26)	-76

Total interviews conducted	**65**
- 3 interviews have not been analysed	-3
	
Total interviews analysed (including 67 elderly people: 5 couples and 57 singles)	**62**

**Table 2:** Range, reliability and sum square (SD) of the measures used

MEASURES	Range	Reliability (Cronbach's α)	Mean score (SD)
Functional status	18–54	0.89	33.9 (7.0)
Subjective perception of health status	0–15	0.83	4.6 (3.7)
Memory	0–9	0.73	3.1 (2.4)
Negative feelings: worries, sadness, anxiety	0–15	0.83	3.1 (3.2)
Feelings of loneliness	0–21	0.79	6.4 (4.3)
Social network	5–25	0.66	16.6 (4.6)
Social participation	4–20	0.76	8.6 (4.5)
Self-care agency	9–45	0.67	30.6 (4.6)

**Table 3:** Acceptance or refusal of admission into a home for the elderly (n = 65)

Characteristics of subjects	Missing Values	Acceptation n = 42	Refusal n = 23	*p-value*
Age (SD)^1^	-	80 (6.5)	83 (4.5)	0.12

Gender^2^	-			0.69
-male		11	5	
- female		31	18	

Living conditions^2^	-			0.98
- living alone		33	18	
- sharing a house with others		9	5	

Housing^2^	1			0.79
- specially adapted house/apartment for senior citizens or elderly people		9	6	
- ground-floor rooms		14	6	
- regular house		18	11	

Waiting period^2^	-			0,39
- 0 – 6 months		12	10	
- 6 – 12 months		12	6	
- > over 1 year		17	6	

Level of physical limitation^1 ^(18–54)^3^	2	35	32	0.14

**Personal perception of health**^1^**(0–10)**	**8**	**3.6**	**6.1**	**0.02**

Loss of memory^1 ^(0–6)	9	3.3	3.1	0.73

Negative feelings^1 ^(0–10)	10	3.3	2.8	0.62

Feelings of loneliness^1 ^(0–14)	8	6.5	5.7	0.45

Social network^1 ^(0–25)	8	16.1	18.2	0.12

Social participation^1 ^(4–20)	1	8.2	9.3	0.36

**Self- care agency**^1^**(9–45)**	**5**	**29.7**	**32.5**	**0.02**

**Urgency of admission**^1^**(1–5)**	**2**	**4.0**	**1.6**	**0.00**

^1.^ ANOVA (one-way)

^2^Chi square

^3^Only 60 subjects answered this part of the questionnaire

**Table 4:** Variables predicting whether elderly persons will accept or refuse an offer of admission into the home for the elderly of their first choice.

	B	*p-value*	OR	CI 95%
Social network	- 0.23	0.03	0.80	0.65 – 0.98
Physical limitations	0.12	0.05	1.13	1.00 – 1.27
